# Familial Hemophagocytic Lymphohistiocytosis: When Rare Diseases Shed Light on Immune System Functioning

**DOI:** 10.3389/fimmu.2014.00167

**Published:** 2014-04-16

**Authors:** Elena Sieni, Valentina Cetica, Yvonne Hackmann, Maria Luisa Coniglio, Martina Da Ros, Benedetta Ciambotti, Daniela Pende, Gillian Griffiths, Maurizio Aricò

**Affiliations:** ^1^Department Pediatric Hematology Oncology, Azienda Ospedaliero-Universitaria Meyer Children Hospital, Florence, Italy; ^2^Pediatric Hematology Oncology Network, Istituto Toscano Tumori (I.T.T.), Florence, Italy; ^3^Cambridge Institute for Medical Research, University of Cambridge Biomedical Campus, Cambridge, UK; ^4^Istituto di Ricovero e Cura a Carattere Scientifico Azienda Ospedaliera Universitaria San Martino-Istituto Nazionale per la Ricerca sul Cancro, Genoa, Italy

**Keywords:** cellular cytotoxicity, natural killer, hemophagocytosis, mutation analysis

## Abstract

The human immune system depends on the activity of cytotoxic T lymphocytes (CTL), natural killer (NK) cells, and NKT cells in order to fight off a viral infection. Understanding the molecular mechanisms during this process and the role of individual proteins was greatly improved by the study of familial hemophagocytic lymphohistiocytosis (FHL). Since 1999, genetic sequencing is the gold standard to classify patients into different subgroups of FHL. The diagnosis, once based on a clinical constellation of abnormalities, is now strongly supported by the results of a functional flow-cytometry screening, which directs the genetic study. A few additional congenital immune deficiencies can also cause a resembling or even identical clinical picture to FHL. As in many other rare human disorders, the collection and analysis of a relatively large number of cases in registries is crucial to draw a complete picture of the disease. The conduction of prospective therapeutic trials allows investigators to increase the awareness of the disease and to speed up the diagnostic process, but also provides important functional and genetic confirmations. Children with confirmed diagnosis may undergo hematopoietic stem cell transplantation, which is the only cure known to date. Moreover, detailed characterization of these rare patients helped to understand the function of individual proteins within the exocytic machinery of CTL, NK, and NKT cells. Moreover, identification of these genotypes also provides valuable information on variant phenotypes, other than FHL, associated with biallelic and monoallelic mutations in the FHL-related genes. In this review, we describe how detailed characterization of patients with genetic hemophagocytic lymphohistiocytosis has resulted in improvement in knowledge regarding contribution of individual proteins to the functional machinery of cytotoxic T- and NK-cells. The review also details how identification of these genotypes has provided valuable information on variant phenotypes.

## Introduction

Defense of an organism against pathogens and cancer is accomplished by the cooperation between different cell types of the human immune system, in particular cytotoxic T lymphocytes (CTL), natural killer (NK) cells, and NKT cells (1). Although these cells use different receptors to identify a target cell, once activated they all employ a similar, highly coordinated machinery that delivers the lethal hit by the secretion of secretory lysosomes (2). Secretory lysosomes in CTL, NK, and NKT cells are lysosome-related organelles that contain perforin, a tetrameric protein, which is able to form pores across lipid bilayers and serine proteases, such as granzymes. Upon release of perforin into the gap formed between the effector and target cell, perforin pores allow granzymes to enter the target cell and to trigger apoptosis by initiation of the caspase cascade (3). Although in this review we will address more specifically some features of the NK cells, because they are suitable for rapid diagnostic assays, it is important to recognize that T-cells share most of these features and have great relevance in most of the human disease related to impairment of this machinery.

Natural killer cells serve as pivotal sentinels within the immune system as they respond quickly to a pathogenic infiltration and alert the host about infections. They are able to recognize and lyse tumor cells and virus-infected cells, even without any previous sensitization. NK cells originate in the bone marrow and are released into the bloodstream upon maturation, thus being able to respond to stimuli such as pathogen molecules, cytokines, or by the interaction with any target cell that expresses ligands for activating NK cell receptors. Since inhibitory receptors recognize the ubiquitously expressed MHC class I molecules, NK cells do not attack healthy cells. On the contrary, NK cell activation occurs when a potential target cell lacks surface MHC class I, as observed in cancer or infection. This concept has been defined as the “*missing self-hypothesis*.” As a result, NK cells behave as fine-tunable effector cells: their regulation may occur at different levels, since the number of receptors which are expressed on their surface may be modified according to the level and type of circulating cytokines. The balance of signals received from activating and inhibitory receptors determines the outcome of NK cell function. Furthermore, an increasing interest is directed to the interaction (*cross-talk*), which occurs between NK cells and other cells engaged in the early phase of inflammatory response (4), which have major implications for the ability of the organism to react to infection and cancer.

Activation and priming of cytotoxic T- and NK-cells induces the formation of secretory lysosomes, which contain lytic molecules. The complex machinery of handling and re-locating these granules became more readable by investigation of human experimental models represented by a congenital immune deficiency in which defective granules activity has a pivotal pathogenic role.

## Hemophagocytic Lymphohistiocytosis

Hemophagocytic lymphohistiocytosis (HLH) is a hyper-inflammatory syndrome observed more often, but not exclusively, in children. Accumulation of reported cases and investigation of consanguineous families allowed to define that the familial form of HLH is indeed a congenital immune deficiency; to date, four subtypes are defined by mutations in the following genes: *PRF1* in familial hemophagocytic lymphohistiocytosis type 2 (FHL2), *UNC13D* in familial hemophagocytic lymphohistiocytosis type 3 (FHL3), *STX11* in familial hemophagocytic lymphohistiocytosis type 4 (FHL4), and *STXBP2* in familial hemophagocytic lymphohistiocytosis type 5 (FHL5). These subtypes will be described below in more details. Furthermore, some patients with other congenital immune deficiencies [X-linked lymphoproliferative disorder, Griscelli syndrome, Chédiak–Higashi syndrome (CHS), and Hermansky–Pudlak syndrome (HPS)] may develop HLH. A fully overlapping clinical picture may be observed, most often following viral infection, in patients with no evidence of genetic defect; these cases have been usually defined as “secondary” or HLH. Patients with HLH are often older than those with underlying genetic defect, but the clinical course may be equally life-threatening. The approach to patients with HLH/FHL is aimed at achieving clinical remission while defining the diagnosis. Definition of FHL has immediate therapeutic implications inasmuch hematopoietic stem cell transplantation (HSCT) is mandatory for FHL, but not for HLH, with the only exception of those cases in which the disease recurs and proves to be familial (and potentially linked to other or unknown genes) or treatment-dependent.

## Familial Hemophagocytic Lymphohistiocytosis

Familial hemophagocytic lymphohistiocytosis is a genetically heterogeneous disorder caused by mutations in genes involved in the secretory lysosome-dependent exocytosis pathway. More than 60 years after the first FHL case was reported, five independent, FHL-causing loci have been identified and the underlying genetic defect has been described for four of them (Table 1). Moreover, we have progressively learned that other immune deficiencies have a clinical picture that may initially lead to a diagnosis of FHL. The genes involved are numerous and some of them are difficult to analyze due to the size of the gene and heterogeneity of mutations. Yet, around 70% of cases are explained by mutations in only two genes: *PRF1* and *UNC13D*, which cause the FHL2 and FHL3 subgroups, respectively (5).

**Table 1:** **Overview of some characteristics of genetic disorders associated with occurrence of hemophagocytic lymphohistiocytosis**.

Subtype	OMIM number	Mutated gene	Locus	Affected protein	Animal model
FHL1	603552		9q21.3–22	Unknown	None
FHL2	267700	*PRF1*	9q21.3–q22	Perforin	Pfn1^−/−^
FHL3	608898	*UNC13D*	17q25.1	Munc13-4	Jinx
FHL4	603552	*STX11*	6q24	Syntaxin 11	None
FHL5	613101	*STXBP2*	19p13	Munc18-2	None
GS2 (Griscelli)	607624	*RAB27A*	15q21	RAB27a	Ashen
CHS (Chédiak–Higashi)	214500	*LYST*	1q42.1–q42.2	LYST	Beige
HPS2 (Hermansky–Pudlak)	608233	*ADTB3A*	5q14.1	AP-3	Pearl
XLP-1	308240	*SH2D1A*	Xp25	SAP	Sh2d1a^−/−^
XLP-2	300635	*XIAP*	Xp25	XIAP	XIAP-ps1^−/−*^

*The XIAP-ps1^−/−^ knockout mouse described in 2009 by Kotevski et al. did not phenocopy the XLP-2 human disease [Ref. (6)].

### Familial hemophagocytic lymphohistiocytosis type 2 (OMIM 603553)

Nearly 15 years after its genetic definition, it is now clear that FHL2 accounts for 20–50% of all FHL cases, depending on the cohort studied (7–9). Mutations in the perforin gene (*PRF1*, OMIM 170280) are responsible for this phenotype (8–10). *PRF1* has three exons: exon 2 and 3 code a 555 amino-acid polypeptide. Over 120 different mutations have been identified to date: 101 missense/non-sense mutations and 21 deletion/insertion mutations (8, 10–17). Since the pathogenic role of some mutations, especially some single amino-acid substitutions, is difficult to assess, their functional characterization is still required. In a recent comprehensive *in silico* analysis of 76 missense mutations in *PRF1*, An et al. used a structural approach to explain the effect of a mutation on the ability of perforin to oligomerize, thereby offering an explanation for the observed defect in cytotoxicity in FHL2 patients (18).

Accumulation of a sufficient number of cases helped to establish connections between specific mutations and particular ethnic groups: c.1122G > A (p.W374X) was found to have a high incidence in Turkish patients (9); mutation c.50delT (p.L17FsX) is very frequent in patients of African-American origin (19); while the c.1090-1091delCT (p.L364fsX) mutation has only been identified in Japanese patients (10).

Mutation c.272C > T (p.A91V) has a particularly high frequency in Southern European population, ranging from 2.5 up to 10% (20, 21). It seems to be present at a very low frequency in African-American subjects and Sub-Saharan Africans, with no reported cases of the polymorphism in Japan, supporting the concept of a Mediterranean origin of the mutation. Although its impact on the protein structure and function has been documented (22, 23), its pathogenic role in FHL has been considered controversial. It is now clear that patients with FHL2 frequently show A91V mutation in combination with another pathogenic mutation; association of A91V with another missense/hypomorphic mutation frequently results in a late-onset of FHL2 (24). Interestingly, in addition to the 106 mutations described in FHL2 patients, other mutations have been associated with different phenotypes (see below).

### Familial hemophagocytic lymphohistiocytosis type 3 (OMIM 608898)

Familial hemophagocytic lymphohistiocytosis type 3 is caused by mutations in the *UNC13D* gene (OMIM 608897), which encodes the 1,090 amino-acids-long protein Munc13-4. Munc13-4 is involved in the priming of secretory granules and their fusion with the plasma membrane, and its loss-of-function consequently impairs the release of perforin and granzyme into the synaptic cleft (25).

Familial hemophagocytic lymphohistiocytosis type 3 covers between 30 and 40% of FHL patients, based on different geographic areas and ethnic groups. The clinical picture of FHL3 patients is undistinguishable from that of FHL2 patients. However, evaluation of patient CTL and NK cells by flow-cytometry showed a clear difference: while FHL2 patients have no perforin but show no degranulation defect, cells from FHL3 patients have normal perforin expression but reduced to absent degranulation (26) (Table 2).

**Table 2:** **Overview of the functional characteristics of genetic disorders associated with occurrence of hemophagocytic lymphohistiocytosis**.

Subtype	Protein expression	NK cell function	Degranulation
FHL1	Unknown	Unknown	Unknown
FHL2	Perforin reduced to absent, detectable by flow-cytometry	Defective killing	Normal
FHL3	Munc13-4 defect, detectable by western blot	Variably impaired	Reduced
FHL4	Syntaxin 11 defect, detectable by western blot	Variably impaired	Reduced
FHL5	Munc18-2 defect, detectable by western blot	Variably impaired	Reduced
GS2 (Griscelli)	Rab27a defect, detectable by western blot	Variably impaired	Reduced
CHS (Chédiak–Higashi)	Lyst defect, detectable by western blot	Variably impaired	Reduced
HPS2 (Hermansky–Pudlak)	AP-3 defect, detectable by western blot	Variably impaired	Reduced
XLP-1	SAP defect, detectable by flow-cytometry	Inhibitory 2B4, defective vs. B-EBV	Normal
XLP-2	XIAP defect, detectable by flow-cytometry (with limitations) and western blot	Enhanced AICD	Normal

*UNC13D* consists of 32 coding exons. To date at least 112 different mutations in *UNC13D* have been reported as a cause of FHL3: 60 missense/non-sense, 25 splicing/regulatory, 25 deletion/insertion mutations, as well as 2 complex gene rearrangements (25–28). After some initial difficulties in recognizing biallelic mutations in patients with FHL3, it became evident that not only exonic mutations, but also variations outside exons and splice sites are a common cause of FHL3 (29). Since then, additional contribution confirmed this issue. In 2011, Meeths et al. described two novel mutations frequently occurring in Northern European populations: the deep intronic mutation c.118-308G > A selectively impairs *UNC13D* transcription in lymphocytes, thus abolishing Munc13-4 expression while the 253-kb inversion affects the 3′-end of the transcript, also abolishing Munc13-4 expression (30). Another deep intronic mutation, c.118-307G > A, was recently reported in a Chinese patient and documented to impair *UNC13D* transcription, possibly by disrupting a transcription factor binding-site or enhancer element (31). Two mutations have been described in specific populations: the c.1596 + 1G > C mutation is described as the most common *UNC13D* mutation in Japan (29) while mutation c.754-1G > C is predominantly found in Korean patients (32). Altogether, the mutation analysis of patients with FHL3 turned out to be far more engaging than that of patients with FHL2.

Recently in a genotype–phenotype study of 84 patients with FHL3 from Italy, Germany, and Sweden, Sieni et al. described that central nervous system (CNS) involvement is more common in patients with FHL3 than with FHL2. Moreover, the combination of fever, splenomegaly, thrombocytopenia, and hyperferritinemia appears to be the most easily and frequently recognized clinical pattern in FHL3, and in association with a defective granule release assay may lead to clinical suspicion of FHL3 (28).

While FHL2 and FHL3 subgroups account for the majority of patients with FHL, additional genetic subgroups have been progressively identified.

### Familial hemophagocytic lymphohistiocytosis type 4 (OMIM 603552)

Familial hemophagocytic lymphohistiocytosis type 4 is caused by mutations in the *STX11* gene (OMIM 605014) (33). This gene consists of two exons and encodes the 287 amino-acid-long SNARE protein Syntaxin 11 (Stx11).

Although original reports of FHL4 were restricted to families of Turkish/Kurdish origin, more recently patients of different origins have been identified with a defect in *STX11*. Despite the initial clustering of cases, at least 12 different *STX11* mutations have been described to date: 5 missense/non-sense mutations, 5 small deletions, 1 small deletion, and 1 gross deletion (34–36). Patients with FHL4 seem to have a later onset and a less severe clinical presentation of the disease compared to FHL2 and FHL3 (37). Mutations in *STX11* have never been associated with variant phenotypes, different from FHL4.

Recently, Sepulveda et al. tried to elucidate the role of *STX11* mutations in the pathogenesis of FHL. They generated a Stx11-deficient (*Stx11*^−/−^) murine model that faithfully reproduced the manifestations of HLH and represented a suitable model for studying FHL4 *in vivo* and the role of Stx11 *in vitro*. By comparing the severity of HLH in *Stx11*^−/−^ mice with that observed in *Rab27a*^−/−^ and *Prf1*^−/−^ mice, they established a correlation between the murine mutants and the age at HLH onset in their human counterparts (38). Furthermore, in a recent report the *STX11* L58P mutation revealed that both the N-terminus and Habc domain of Stx11 are required for binding to Munc18-2, implying similarity to the dynamic binary binding of neuronal syntaxin 1 to Munc18-1 (39).

### Familial hemophagocytic lymphohistiocytosis type 5 (OMIM 613101)

Familial hemophagocytic lymphohistiocytosis type 5 is due to mutations in *STXBP2* (also named *MUNC18-2*; OMIM 601717) (40, 41) and has been reported to account for up to 20% of cases with FHL in the German series (42). Since 2010, 40 different mutations of *STXBP2* have been described: 15 missense/non-sense, 10 splicing/regulatory, and 15 deletion/insertion mutations (41, 43–46). FHL5 does not appear to be restricted to a specific geographic region.

In contrast to what was observed for the comparison between FHL2 and FHL3, some clinical presentations of FHL5 seem to be different from other classical manifestations of FHL. Gastrointestinal symptoms, such as chronic diarrhea, gastro-esophageal reflux, and abdominal pain are present in a significant number of patients. Renal tubular dysfunction was also observed in one patient. This could be explained by an impaired expression and function of Munc18-2/STXBP2 protein in cells other than cytotoxic lymphocytes, including intestinal and renal epithelium (47). The defect caused by insufficient function of STXBP2 protein in the neutrophils is associated with defective mobilization of the granules. As a result, the cell is unable to kill bacteria; insufficient clearance of *E. coli* might be one or the main reason for the frequency of gastrointestinal symptoms in patients with FHL5 (42, 46–48).

Since platelets contain syntaxin-binding proteins with non-redundant functional roles, platelets from FHL5 patients have defective secretion, with decreased Munc18-2 and Stx11 levels. These data demonstrated a key role for Munc18-2, perhaps as a limiting factor, in platelet exocytosis, suggesting that it regulates Stx11 (49). These data together with those of Pagel et al. (41) suggest that, although bleeding histories may be too variable to be a sufficient diagnostic criteria, platelet function assays may be worth investigating in patients with FHL.

## Additional Genetic Immune Deficiencies Associated with HLH

In addition to the described four subgroups of FHL, in which HLH is usually the primary manifestation, a few additional genetic conditions may cause a clinical syndrome largely overlapping that of FHL but in which additional, distinctive clinical features occur.

### X-linked lymphoproliferative disease 1 (Duncan disease, OMIM 308240)

X-linked lymphoproliferative disease 1 (XLP-1) is a rare congenital immunodeficiency caused by mutations in *SH2D1A* (Xq25), the gene encoding the signaling lymphocyte activation molecule (SLAM)-associated protein (SAP) (50, 51).

In immune-competent individuals Epstein–Barr virus (EBV) causes infectious mononucleosis, a common, usually self-limited disease. In XLP-1, the lack (or dysfunction) of SAP causes the selective inability to control infection by EBV, a γ-herpes virus that infects B-cells (52–55). Several immunological defects have been identified, including defective NK and CD8^+^ T-cell-mediated cytolytic responses against EBV-infected cells, which lead to B-cell accumulation and persistence of reactive inflammatory responses (56). In the absence of SAP, 2B4 receptor (member of SLAM family), when engaged by its ligand CD48, delivers inhibitory instead of activating signals (53). It has been recently demonstrated that in XLP-1 NK cells the co-engagement of 2B4 with different activating receptors inhibits NCR, CD16, and activating KIRs, characterized by ITAM-dependent signaling pathways. In contrast, the 2B4 dysfunction does not affect the activity of DNAM-1 and NKG2D triggering receptors. Thus, while CD48^+^ B-EBV and lymphoma B-cells devoid of NKG2D and DNAM-1 ligands were resistant to lysis, the preferential usage of these receptors allowed XLP-1 NK cells to kill lymphomas that expressed sufficient amounts of the specific ligands (57). Better knowledge of the underlying dysfunction could be turned into a diagnostic tool. Patients with XLP-1 may present with different phenotypes: fulminant mononucleosis, B-cell lymphoma, lymphoproliferation, and dysgammaglobulinemia (56, 58, 59), but also with HLH (60). Thus, differential diagnosis is relevant. To this issue, immunological screening for intra-cytoplasmic SAP expression and rapid assays to examine 2B4 receptor function, which is inhibitory instead of activating in *SH2D1A* mutated patients (53, 57), may be applied (Manuscript in preparation).

In our review of the literature, 100 *SH2D1A* mutations were found: 46 missense/non-sense, 14 splicing, 2 regulatory mutations, 9 small deletions, 6 small insertions, and 23 gross deletions (50–58, 61). Interestingly, this gene is characterized by a high number of deletions including the entire gene. Intronic mutations have also been described affecting *SH2D1A* transcription but not mRNA splicing, and leading to markedly reduced level of SAP protein (61). Thus, the strategy of mutation analysis of this gene must be designed to include these possible variants.

### X-linked lymphoproliferative syndrome type 2 (OMIM 300635)

A subset of patients with an XLP-like phenotype was recently found to have mutations in *BIRC4*, the gene encoding the X-linked inhibitor of apoptosis protein (XIAP) and has been linked to another subgroup, named X-linked lymphoproliferative syndrome type 2 (XLP-2) (62).

X-linked inhibitor of apoptosis protein is an essential ubiquitin ligase for pro-inflammatory signaling downstream of the nucleotide-binding oligomerization domain containing (NOD)-1 and -2 pattern recognition receptors. Recently, the XIAP baculovirus IAP repeat (BIR2) domain was recognized as a hotspot for missense mutations in XLP-2. XLP-2-BIR2 mutations severely impair NOD-1/2-dependent immune signaling in primary cells from XLP-2 patients and in reconstituted XIAP deficient cell lines. XLP-2-BIR2 mutations abolish the XIAP–RIPK2 interaction resulting in impaired ubiquitylation of RIPK2 and recruitment of linear ubiquitin chain assembly complex (LUBAC) to the NOD-2-complex. These new findings document that impaired immune signaling in response to NOD-1/2 stimulation is a general defect in XLP-2 and demonstrate that the XIAP BIR2–RIPK2 interaction might be even targeted pharmacologically to modulate inflammatory signaling (63).

In a comparison of the clinical phenotypes associated with XLP-1 and XLP-2, EBV infection was the common trigger of HLH in 92% of XLP-1 and 83% of XLP-2. HLH (XLP-1, 55%; XLP-2, 76%) and hypogammaglobulinemia (XLP-1, 67%; XLP-2, 33%) occurred in both groups, although with different proportions. Survival rates and mean ages at the first HLH episode did not differ for both groups, but HLH was more severe with lethal outcome in XLP-1. Only XLP-1 patients developed lymphomas while XLP-2 patients preferentially displayed chronic hemorrhagic colitis, recurrent splenomegaly often associated with cytopenia and fever (64).

In a similar study, Marsh et al. reported an early disease onset during infancy for XLP-2-linked HLH and a high relapse rate; however, this seemed to occur even in the absence of an EBV infection. Some XLP-2 patients develop hypo/dysgammaglobulinemia resulting from humoral immune system derangement. Intriguingly, and in contrast to XLP-1, XLP-2 was never associated with common variable immunodeficiency (64).

In a third consortium review of 25 patients, the majority initially presented with manifestations other than HLH, such as Crohn-like bowel disease (*n* = 6), severe infectious mononucleosis (*n* = 4), isolated splenomegaly (*n* = 3), uveitis (*n* = 1), periodic fever (*n* = 1), fistulating skin abscesses (*n* = 1), and severe Giardia enteritis (*n* = 1). Subsequent manifestations included celiac-like disease, antibody deficiency, splenomegaly, and partial HLH. Screening by flow-cytometry identified 14 of 17 patients in this cohort (65). Given these clinical differences, XIAP deficiency must be considered in a wide range of clinical presentations. It has recently been suggested that XIAP deficiency would be better classified if defined as an X-linked subtype of FHL, rather than as a second type of XLP (64).

To date, 41 mutations are known in *BIRC4*: 20 missense/non-sense, 2 splicing mutations, 2 regulatory mutations, 16 deletions/insertions, and 1 complex rearrangement (62, 64, 66, 67). The phenotypic differences may be the result of differences in the molecular basis of each disease. However, neither genotype, nor protein expression, nor results from cell death studies were clearly associated with the clinical phenotype.

Although HSCT remains the milestone for cure of FHL, some discrepancy recently emerged in the outcome of patients with XLP-2. In an international survey of 19 patients, 7 received myeloablative (MAC) regimens, 1 received an intermediate-intensity regimen, and 11 received reduced intensity conditioning (RIC) regimens predominantly consisting of alemtuzumab, fludarabine, and melphalan. The probability of survival was very low in the MAC group, with all but one patient dying from transplantation-related toxicities (especially veno-occlusive disease and pulmonary hemorrhage); otherwise, 55% of those who received RIC survived at a median of 570 days after HSCT. The probability of surviving in the RIC was enhanced by disease inactivity at the time of HSCT. Based on these findings, MAC regimens should not be used for patients with XIAP deficiency. The reason may be connected with the loss of XIAP anti-apoptotic functions in XLP-2 patients (68).

### Immunodeficiencies associated with HLH and partial albinism

At present three syndromes are known to cause HLH and manifest with partial albinism.

### Chédiak–Higashi syndrome (OMIM 214500)

Around 85% of CHS patients develop HLH in the first decade of life. They show markedly defective cytotoxicity of both NK cells and CTL. The genetic defect is caused by mutations in the *LYST* gene (69), which encodes a 3,801 amino-acid protein. Each clinical manifestation of CHS (albinism, bleeding tendency, recurrent bacterial infections, neurologic dysfunction, and HLH) (70, 71) is associated with a defect of a specific cell type and the formation of enlarged lysosomes in these cells.

The presence of giant inclusion bodies of lysosomal origin in a variety of granule-containing cells, including hematopoietic cells and melanocytes, has thus become the hallmark of the disease (72). This feature together with HLH and oculo-cutaneous albinism can address clinical suspicion toward CHS and direct molecular analysis to *LYST* sequencing. In our revision of the available literature, a total of 56 mutations were found to be reported at the time of writing: 23 missense/non-sense, 4 splicing, 20 small deletions, 8 small insertions, and 1 gross deletion. As expected, disruptive mutations correlated with the severe form of the disease (73). The wider diffusion of mutation analysis, despite the big size of the gene, provided an increased number of reports from different geographic areas during the last few years, confirming that CHS has no ethnic or geographic boundaries.

A rare neurologic disorder, named hereditary spastic paraplegia (HSP) and characterized by leg spasticity, weakness, hyperreflexia, and additional neurological symptoms, was recently reported in two adult siblings with HSP and homozygous *LYST* pathogenic mutation. Large peroxidase-positive granules were observed in both patients’ granulocytes, while pigment deficiency, immune deficiency, and bleeding tendency were not observed. This example illustrates nicely how the clinical spectrum of CHS may be much broader than recognized at present (74).

### Griscelli syndrome type 2 (OMIM 607624)

Griscelli syndrome is a rare autosomal-recessive disorder characterized by partial oculo-cutaneous albinism and HLH (75). Among GS subtypes, only patients with type 2 develop HLH. Mutations causing Griscelli syndrome type 2 (GS2) were mapped to the *RAB27A* gene, which is composed of 5 coding exons and encodes for a 221 amino-acid protein that belongs to the superfamily of small Rab GTPase. CTLs and NK cell activity defect results from the inability of cytotoxic granules to dock to the plasma membrane whereas hypopigmentation is accounted for by a defective release of melanosomes from melanocyte dendrites. Change in phagosomal function and antigen cross-presentation of Rab27a-deficient dendritic cells has been reported *in vitro*. In the mouse model (ashen mice), the dendritic cells are unable to perform a sufficient antigen cross-presentation (76).

To date 34 *RAB27A* mutations are known: 15 missense/non-sense, 4 splicing, and 15 deletions/insertion. In 2010, Meeths et al. sequenced *RAB27A* in patients diagnosed as HLH and found one mutated family (77). Since the clinical picture of the two syndromes is indistinguishable, they concluded that the diagnosis of GS2 may be overlooked, particularly in fair-haired patients with hemophagocytic syndromes.

### Hermansky–Pudlak type 2 (OMIM 608233)

The term HPS encompasses nine different human autosomal-recessive genetic disorders, sharing partial oculo-cutaneous albinism and bleeding disorders (78, 79). Furthermore, patients with Hermansky–Pudlak type 2 (HPS2) also show an increased susceptibility to infections, resulting from both congenital neutropenia and impaired cytotoxic activity. Mutations of the gene encoding the β-3A subunit of *adaptor protein-3* (AP-3) complex are the cause of HPS2 (80). To date, 20 mutations are known in this gene and are associated with HPS2: 7 missense/non-sense, 1 splicing, 11 deletions/insertion, 1 complex rearrangement, and a chromosome 5 inversion that disrupts the gene sequence (81). To date, only one HPS2 patient has been reported who developed HLH. However, since this patient also carried a potentially contributing heterozygous RAB27A mutation, the risk to develop HLH in HPS2 remains unclear (82). The pearl mouse model of HPS2, upon infection with lymphocytic choriomeningitis virus, developed all the key features of the disease, which yet was only transient (82). In a cohort of 22 HPS2 patients, only one additional patient with HLH was identified; two developed incomplete, transient HLH-like episodes, although the cytotoxicity or degranulation capacity was impaired in all 16 patients tested (83). Although future reports might clarify the genotype–phenotype correlations, the risk for HLH in HPS2 appears lower than in Griscelli or CHS (79, 82).

## Clinical Picture of FHL

Most patients with FHL are brought to the attention of the pediatrician because of long-lasting fever, which does not respond to antibiotic therapy. Physical examination usually shows hepatosplenomegaly (84); up to 30% of cases also show neurological abnormalities, from irritability to cranial nerve palsy or seizures. Cerebrospinal fluid analysis shows alterations in more than half of patients, with pleocytosis, increased protein, or both. Several studies have defined the pattern of alterations evident at neurologic imaging: parenchymal atrophy, diffuse abnormal signal intensity in the white matter on T2-weighted images, focal hyperintense lesions, delayed myelination, or parenchymal calcification (85, 86).

Characteristic bio-markers are elevated ferritin, triglycerides, α-chain of the soluble interleukin-2 receptor (sCD25), and low fibrinogen. Hemophagocytosis by activated macrophages (which became popular by being included in most of the different names used for this disease since its original report in 1952) may be lacking at initial bone marrow examination. This negative finding does not preclude the diagnosis of HLH, as well as the presence of hemophagocytosis alone does not make the diagnosis of HLH, and should be considered supportive evidence only. Thus, the relevance of hemophagocytosis in the set of diagnostic criteria might even be questionable. Additional findings in a minority of patients are: lymphadenopathy, icterus, rash, edema, high levels or transaminases, bilirubin, and lactate dehydrogenase. Accumulation of cases allowed to characterize unusual presentations: acute liver failure or isolated CNS involvement may be observed. This led to the practice to consider HLH in the differential diagnosis of patients scrutinized for possible liver transplant (87). Identification of high levels of selected cytokines provided a likely explanation for some clinical features: fever is induced by IL-1 and IL-6; pancytopenia results from high levels of IFN-γ and TNF-α and also from hemophagocytosis; hypertriglyceridemia results from the inhibition of lipoprotein lipase by TNF-α; ferritin is secreted by activated macrophages, also responsible for the high levels of plasminogen activator, which cause high plasmin levels and hyperfibrinolysis. The picture of the contribution of the individual cytokines and chemokines is entirely under thorough evaluation by several investigators.

## Diagnostic Strategy

To facilitate an initial approach to the disease, in 1994 the Histiocyte Society defined a set of diagnostic criteria; they were subsequently revised in 2004 (Table 3) (88). Nevertheless, diagnosing FHL still represents a challenge for the pediatrician. Although most of the cases develop the disease when they are very young, about 20% of cases only present once the subject is older than 2 years (76); later onset, including the young adult age, is increasingly reported (89–92). The information of parental consanguinity may be useful but is expected in no more than 25% of cases. The family history may include the early death of a sibling with undefined cause, or diagnosed as “lymphoma or infection.” Evidence of partial albinism, or “light hairs” is not frequent but, when present, turns to be very informative.

**Table 3:** **Revised diagnostic guidelines for hemophagocytic lymphohistiocytosis (HLH)**.

The diagnosis of HLH can be established if either 1 or 2 below are fulfilled
1. A molecular diagnosis consistent with HLH
2. Clinical and laboratory criteria for HLH fulfilled (5/8 criteria below)
Fever
Splenomegaly
Cytopenia (affecting ≥2 of 3 lineages in peripheral blood)
Hemoglobin <9 g/dl (in infants <4 weeks: Hb <10 g/dl)
Platelets <100 × 10^9^/l
Neutrophils <1.0 × 10^9^/l
Hypertriglyceridemia and/or hypofibrinogenemia
Fasting triglycerides ≥3.0 mmol/l
Fibrinogen ≤1.5 g/l
Hemophagocytosis in bone marrow or spleen or lymph nodes
Low or absent NK cell activity
Ferritin ≥500 μg/l
Soluble CD25 (i.e., soluble IL-2 receptor) ≥2,400 U/ml

*Supportive evidences are cerebral symptoms with moderate pleocytosis and/or elevated protein, elevated transaminases and bilirubin, LDH*.

**Figure 1:**
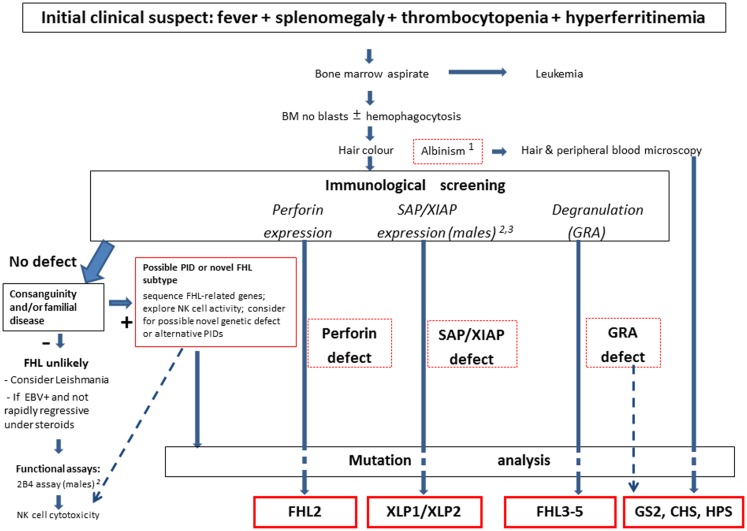
**Overview of the current diagnostic approach to FHL**. The clinical suspect is raised by a few elements, which may trigger the use of the functional laboratory screening at flow-cytometry. Mutation analysis remains the gold standard for confirmation of the diagnosis, with therapeutic implications. Notes: 1. Some patients with pigment dilution syndromes (GS, CHS, or HPS) may have gray or very blond hairs, not necessary albino discoloration. 2. Although lack of SAP expression at flow-cytometry is invariably associated with XLP-1, in rare cases patients with XLP-1 may have residual, reduced, or even normal SAP expression; thus the 2B4 assay may be used to discriminate between normal (activating 2B4) or defective (inhibitory 2B4) SAP function. 3. The use in flow-cytometry of currently available anti-XIAP mAb does not provide fully satisfactory results, while it is more suited for western blot analysis. Thus mutation analysis may be necessary to rule out the diagnosis in the presence of suggestive clinical picture.

The set of alterations described above, however are not specific. Thus, in many cases leukemia is suspected at first but quickly ruled out by bone marrow examination, which shows hemophagocytosis in about one-half of all cases. Similarly, none of the biochemical abnormalities described earlier are exclusive to HLH. Investigators wonder if these criteria might be simplified. The evidence of defective NK cell activity, although very suggestive for the diagnosis, is laborious and restricted to a limited number of laboratories also due to the usual need for radionuclide reagents. The real role of hemophagocytosis is repeatedly questioned. To this issue, we observed that the combination of fever, splenomegaly, and thrombocytopenia, in the absence of leukemia, represents the starting point to suspect FHL (28). When this is associated with hyperferritinemia, the clinician may consider it as a sufficient basis to suspect HLH and thus address the diagnostic work-up already within a few hours from admission (Figure 1). The level of D-dimers is usually abnormal even when INR/PTT is normal. The possible contribution of additional parameters, such as plasma levels sCD25, sCD163, neopterin, and IFN-γ appears increasingly convincing (86).

As in many other immune deficiencies, common pathogens, especially viruses, may represent an excessive challenge for the child with FHL and thus trigger the onset of the disease. It is very important to remember that patients with visceral leishmaniasis may present with a very similar clinical picture (93), but especially in non-endemic areas this may not be readily included in the differential diagnosis. Unfortunately, patients with undiagnosed leishmaniasis have been diagnosed and then treated as FHL, with major consequences. Thus, the use of polymerase chain reaction (PCR) for investigation of selected infectious agents, including EBV, cytomegalovirus (CMV), and Leishmania appears to be recommended.

If a patient shows the set of initial parameters that match the diagnostic hypothesis, such as fever, splenomegaly, thrombocytopenia, and elevated ferritin, the attending physician should approach an immunology laboratory, which is able to perform a functional screening. Impaired NK cell cytotoxicity, measured as lysis of K652 cells by peripheral blood lymphocytes in a standard chromium release assay, became the hallmark of FHL. However, the variability of NK cell percentages in PBL among different individuals and the use of radioactivity present major limitations to get precise and standardized results in different laboratories. The use of the NK cytotoxicity assay is useful when it shows reduced or absent activity, but normal NK function assay should not definitely exclude the diagnosis of FHL.

Thus, a set of tools for the screening of FHL and other genetic immune deficiencies associated with HLH has been developed. A deficient intra-cytoplasmic expression of perforin by NK cells can identify patients with FHL2 (14). Furthermore, defective intracellular expression of SAP and XIAP are associated with XLP-1 and XLP-2, respectively. For patients with normal findings at the above described assays, Marcenaro et al. have originally demonstrated that surface CD107a expression represents a rapid tool for identification of patients with degranulation defect (25). CD107a (LAMP1) lines the lysosome containing perforin and granzymes to prevent the NK and CTL itself from damage. After activation, the granules move to the synapse with a fine-tuned process of polarization and docking to the cell membrane. After the degranulation into the synapse, CD107a can be identified on the surface of NK and CTLs. Mutations of genes that produce proteins necessary for this secretory mechanism result in the absence of CD107a measured by flow-cytometry. In FHL2, perforin is low or absent but degranulation still takes place. Thus it is important to measure perforin expression before the degranulation assay is done. This is true as well for XLP-1 and XLP-2 which are not associated with failure of degranulation, and these proteins should be measured at least in male patients with suspected genetic HLH. It is important to note that although a minority of patients with a clinical diagnosis of secondary HLH may have an abnormal resting NK cell degranulation, none shows abnormal degranulation using interleukin-2 (IL-2)-activated NK cells (94).

A degranulation defect, which is present in the majority of FHL cases, was first documented in patients with FHL3 (95), and rapidly became the standard for their identification. Thereafter, additional reports confirmed this finding also in patients with FHL4, FHL5, GS2, CHS, and HPS2 (94). In patients with a high probability for genetic defect, in whom flow-cytometry screening of relevant molecules was normal, assessment of cytotoxic activity appears mandatory. Repeated evidence for complete or partially defective killing and/or degranulation should be taken as strong support for the diagnosis of primary HLH (94). In addition, the clear evidence of a functional defect can guide therapy even before a mutation is identified.

Figure 1 describes an overview of our current diagnostic strategy for FHL. In summary, these assays provide an initial confirmation of the clinical diagnosis, and direct the mutation analysis, which yet remains the gold standard for the diagnosis. In 80% of familial HLH cases the genetic error can be identified by sequencing. Knowledge of the genetic background has a great impact on the treatment of the patient: it supports indication towards HSCT, helps with the selection of a matched donor, and represents a useful tool to counsel the family, and to offer prenatal diagnosis if requested.

## Treatment of FHL

Historical reports showed that patients with HLH have an exceedingly high risk of mortality within weeks (84), unless an appropriate treatment is started promptly. According to the patient condition, the treatment may be started even before the results from some diagnostic studies become available. The immediate aim of therapy is to suppress the hyper-inflammatory state and to kill not only the exuberant lymphocytes, but also the pathogen-infected antigen-presenting cells. This removes the stimulus, thus breaking the vicious loop of continuous but ineffective activation of cytotoxic cells. The first international cooperative study HLH94 set the combination of dexamethasone and etoposide as the standard of care (88, 96). This strategy brings most patients into a state of disease control within 4–8 weeks. This may buy enough time for further diagnostic tests. For those patients with evidence of a genetic defect, HSCT is strongly recommended, it being the only treatment that can cure FHL to date. Yet, this is not an easy and universal solution. Although recent advances in transplantation procedures and supportive therapy minimize the transplant-related mortality, this is particularly true for 20% of the patients who have a matched familial donor, the procedure remains risky and by no means a guarantee for survival. For the remaining cases, a matched unrelated, or partially matched familial donors, or cord blood units, are the possible alternative sources for HSCT. Since treatment related mortality in this setting remains a big threat, it is extremely important that a recommendation for HSCT is correctly defined. The use of RIC for HSCT provided a significant improvement especially by reducing the unacceptably high level of toxicity connected with the use of ablative regimens (and in particular the veno-occlusive disease resulting from exposure to busulphan) (97, 98). Although the use of RIC may be associated with incomplete donor chimerism, the use of anti-thymoglobulin (ATG) or alemtuzumab and fine tuning of its timing has considerably improved the treatment results (99). Moreover, as in other constitutional or acquired immune disorders, mixed chimerism may turn out to be sufficient to control the disease by replacing the defective function (100, 101).

For patients with normal function at initial screening, the current treatment strategy also suggests to allow a chance for treatment withdrawal after disease resolution in order to avoid, potentially unnecessary, HSCT. For patients with refractory disease or with disease reactivation, which appear unable to remain disease-free in the absence of chemo-immunotherapy, transplantation will be considered even in the absence of a documented genetic defect.

One major problem in the cure of patients with FHL remains the persistent, unacceptably high rate of mortality in the early, pre/transplant phase. In 1993, Stephane et al. (102) reported an ATG-based regimen associated with a more rapid response rate, explained by its direct attack of CTL, which are thought to drive the disease process. These data were updated and expanded by the same group in 2007 (103). Unfortunately, this regimen may lead to shorter remissions and higher reactivation rates, when compared to the standard etoposide-based regimen. Because these two regimens have complementary strengths (and weaknesses), a pilot study has been designed to adopt a hybrid regimen, which combines initial ATG with subsequent weekly doses of etoposide. This approach has been adopted in a pilot study run in parallel in Europe and in the USA, under the name EURO-HIT-HLH (EudraCT Number 2011-002052-14) and HIT-HLH (ClinicalTrials.gov Identifier: NCT01104025), respectively.

Furthermore, data derived from the animal model suggest that blocking the IFN-γ activity may induce disease control without cytoreduction. Thus, the feasibility and the therapeutic potential of a novel human anti-IFN-γ agent denominated NI-05-01 is currently explored in a phase II study run on an international setting (EudraCT Number 2012-003632-23).

Finally, the hypothesis to cure patients with FHL using gene transfer is supported by its monogenic origin. To this issue, the recent report that gene transfer corrects the cellular and humoral defects in SAP(^−/−^) mice provides proof of concept for gene therapy in XLP-1 (104).

## Macrophage Activation Syndrome

Children and adults with autoimmune diseases, especially systemic onset juvenile idiopathic arthritis (s-JIA), may develop a clinical syndrome closely resembling, or even overlapping, HLH. This condition has been repeatedly defined as “macrophage activation syndrome” (MAS). Specific diagnostic criteria for MAS complicating JIA have been developed. In particular, persistent continuous fever ≥38°C, falling leukocyte count, falling platelet count, increased liver enzymes, hyperferritinemia, falling erythrocyte sedimentation rate, hypofibrinogenemia, hypertriglyceridemia, and evidence of hemophagocytosis in the bone marrow characterize severe MAS (105). About 7% of patients with JIA develop life-threatening MAS (105–107). Although treatment with immunoglobulin and cyclosporine A has been defined as the current standard for MAS, in some patients a more aggressive therapy, similar to that of HLH, may be required. Several cases of s-JIA-associated MAS dramatically benefiting from the IL-1 receptor antagonist, anakinra, after inadequate response to corticosteroids and cyclosporine A have now been reported (108). It is noteworthy that, although most patients with MAS have normal or reduced NK cell function, they show reduced expression of perforin or SAP, and heterozygous mutations in one FHL-related genes (109–111), thus launching a bridge between FHL and the pathogenic mechanisms of MAS. Attempts to harmonize the nomenclature between HLH and MAS are ongoing in cooperation by pediatric hematologists and rheumatologists.

## Variant Phenotypes Recognized in Association with Partial Cytotoxic Defects

Whereas the complete cytotoxic defect, due to biallelic disruptive mutations in one of the FHL-related genes, leads to full-blown FHL, with the typical and rapidly fatal course, the clinical impact of a less complete or partial defect in this pathway remains yet to be clarified.

However, over the last years, reports of later onset of FHL up to adult age and associations between monoallelic mutations of the FHL-related gene and conditions other than FHL suggest that, although far from causing the full-blown picture of FHL, partial insufficiency of the cytotoxic machinery could pave the way to alternative phenotypes.

### Later onset of FHL

The age at diagnosis of FHL is usually very young, with a peak incidence between 1 and 6 months of age (84). Nevertheless, over the years sporadic cases of FHL in older patients have been reported, pointing to unexpected later onset. In 2001, Allen et al. reported four familial cases of HLH diagnosed on a clinical basis, at an age comprised between 9 and 17 years (89). Soon after, Clementi et al. described the first adult cases in two siblings developing FHL2 at 22 and 21 years of age (91). This was followed by other reports of FHL2 in older children or adolescents of different ethnic origins: one 7-years-old patient from Russia, two North American patients of 8 and 10 years (17), one 10-year-old Turkish patient (9), three Japanese patients of 7–12 years (112), and more recently, three Columbian patients aged 5–12 years (113). The oldest patients reported to date are a Spanish man of 49 years, homozygous for *PRF1* A91V (114), and a 62-year-old Japanese man, compound heterozygous for a *PRF1* missense and a non-sense mutation (115). In 2011, Zhang et al. described 10 adult patients with FHL due to biallelic mutations in *PRF1* (*n* = 7), *MUNC13-4* (*n* = 2), and *STXBP2* (*n* = 1) (24), showing that not only FHL2 could present later in the life.

Despite these sporadic reports, adult patients are still at most considered as affected by the “secondary” non-genetic form of HLH, which has several implications in treatment and outcome. Thus, with the aim to raise the attention of adult specialists to this rare disease we recently described our experience in diagnosing FHL in subjects older than 18 years in Italy. Out of the 197 patients referred to the Italian Registry of HLH in which a genetic defect in FHL-related genes had been identified, 11 (6%) were older than 18 years with a median age of 23 years (range, 18–43 years). FHL2 was the most frequent subtype (*n* = 6) with A91V the most frequent single mutation. The other genetic diagnoses were: FHL3 (*n* = 2), FHL5 (*n* = 1), XLP-1 (*n* = 2). Only one-half of these patients presented with the full-blown picture of HLH, while the other half had atypical manifestations at the onset, which brought them often to the attention of non-hematological specialists. This led to frequent delay in diagnosis and treatment. The clinical course was aggressive and led to early death in 8 of 11 (72%) patients supporting the indication to treat these patients until HSCT (92).

To our knowledge, all the patients with later onset of FHL reported to date have at least one missense mutation, while none had biallelic disruptive mutations. This is in keeping with the results of the genotype–phenotype studies of FHL, which established a close correlation between biallelic disruptive mutations and early age at the onset of FHL (10, 28). Thus, we can speculate that in the presence of hypomorphic genetic defects, residual protein allows some level of NK- and T-cell function, sufficient to cope with common infectious agents, the usual triggers of FHL, at least for several, or even many years. This is also described in a gene-expression profiling study showing that patterns of up- and down-regulated genes separated patients with “late-onset” and “relapsing” forms of FHL from patients with an “early onset and rapidly evolving” form of the disease (116).

### Association between monoallelic mutations of FHL-related genes and conditions other than FHL

Cellular cytotoxicity by NK cells and CTLs plays a central role in immune surveillance and tolerance through granule-dependent exocytosis pathway or death-receptor pathway. This gave rise to association studies aimed at investigating if genes involved in programed cell death of lymphocytes could contribute to cancer and autoimmune susceptibility (117).

#### Lymphoma

The description of the association between different kind of lymphoma and HLH (118, 119) has been followed by studies reporting the link between monoallelic *PRF1* mutations and lymphomas (120–124). Lymphoma or HLH was reported in two siblings with *PRF1* mutations (91), and then autoimmune lymphoproliferative syndrome and lymphoma in a patient with heterozygous *Fas* and *PRF1* mutations (122). Later on, Clementi et al. described four patients with Hodgkin or non-Hodgkin lymphoma who had biallelic perforin mutations and four additional patients with monoallelic *PRF1* mutations (123). A possible predisposing role of *PRF1* variants for ALCL was later established, with the finding of mutations in 27% of children with this subtype of lymphoma (124). We recently decided to extend this study to a larger, unselected population of children with ALCL, and to investigate them for even other FHL-related genes. In line with the previous data, 23 of the 84 (27%) children with ALCL were found to have monoallelic mutations in one of three genes: 21 patients (25%) carried a total of 10 different mutations of *PRF1*, 2 additional patients had missense mutations of the *UNC13D* gene, but no mutations were found in the gene *SH2D1A*. The observation that *PRF1* is involved in a quarter of patients with ALCL suggests a correlation between insufficient cellular cytotoxicity and development of ALCL. Less frequent involvement of *UNC13D* may suggest that subjects heterozygous for mutations in this gene may have others escape mechanisms to prevent lymphoma. *SH2D1A*, the XLP-1 gene, is not related to childhood ALCL (125). Yet, the observation that 5 of 158 males presenting with B-cell NHL (3.2%) had *SH2D1A* mutations raises the issue of prospective screening for XLP-1 in males with B-cell lymphoma (126).

As an extension of their previous association study on lymphoma, Santoro et al. in 2005 sequenced *PRF1* in 100 children with acute lymphoblastic leukemia detecting the most frequent *PRF1* mutation (A91V) in 12/100 patients and 5/127 controls (*p* = 0.014) (19). A similar study of the Cincinnati group, on a much larger cohort, confirmed this association only for *BCR-ABL* positive acute lymphoblastic leukemia (127).

The role of *PRF1* mutations in the predisposition to cancer has been addressed repeatedly especially by the Australian group. In a first study they analyzed 23 individuals with biallelic *PRF1* mutations whose onset of FHL was delayed or abolished and found that 11/23 presented as the primary clinical illness with B- or T-cell lymphoma or acute or chronic leukemia (128). In the following study of 81 European or Maghrebian families in which hematological malignancies had been diagnosed, they found that 3.7% had a *PRF1* variant. The A91V mutation and the N252S polymorphism were already known, while the novel A211V missense substitution was observed in two related Tunisian patients. However, they also report that the lytic function of perforin was not affected by over-expression of mutated *PRF1* in rat basophilic leukemia (119). Subsequently they examined the effect of perforin deficiency in four models of mouse B-cell lymphomagenesis. Perforin was shown to act as a suppressor of B-cell malignancies characteristically driven by v-Ablor bcl-2, whereas Mlh loss cooperated in accelerating spontaneous B-cell lymphomas characteristic of pfp loss. No protective role for perforin was observed in the more aggressive E-myc model of B-cell lymphoma. These transgenic models have allowed to pinpoint the role of perforin in surveillance of B-cell lymphomagenesis (129).

#### Autoimmune lymphoproliferative syndromes

Following the previous identification of a heterozygous mutation of *PRF1* in an autoimmune lymphoproliferative syndromes (ALPS) patient, in 2006 Clementi et al. analyzed 14 ALPS and 28 Dianzani autoimmune lymphoproliferative disease (DALD) cases and found a different amino-acid substitution in 2 of 14 ALPS and 6 of 28 DALD. This suggested that variations in genes involved in the cytotoxicity of CTL and NK cells may also influence ALPS and DALD presentation (130). To assess the potential role of *UNC13D* gene in the susceptibility to ALPS and DALD, Aricò et al. recently sequenced *UNC13D* in 21 ALPS and 20 DALD patients detecting 4 rare missense variations in 3 heterozygous ALPS patients. Transfection of the mutant cDNAs into HMC-1 cells showed that they decreased granule exocytosis, compared to the wild-type construct. These data suggested that rare loss-of-function variations of *UNCD13D* are risk factors for ALPS development (131).

Boggio et al. analyzed *SH2D1A* in ALPS and DALD patients based on the observation of Komori et al. (132) that suggested an opposite epistatic relationship between the Fas and SAP defects in mice. They found that ALPS and DALD patients displayed an increased frequency of the 346T single nucleotide polymorphism (SNP) in *SH2D1A* causing a loss of the −346°C methylation site and correlating with increased SAP expression and decreased IFN-γ production. Based on this finding they suggested high SAP expression promotes development of ALPS and DALD in human (133).

#### Multiple sclerosis

The screening of *PRF1* mutations in 1,156 patients with multiple sclerosis showed a higher frequency of monoallelic or biallelic A91V mutations compared to the control group (1,788 subjects) (134).

#### Arthritis and other rheumatologic disorders

Several groups have reported decreased levels of perforin in CTLs and in particular NK cells, along with decreased NK cell function in patients with s-JIA (109, 110). The first case of association of s-JIA and FHL-related genes was reported by Hazen et al. in 2008, who described an 8-year-old girl with s-JIA and hemophagocytosis, without complete criteria of MAS, who was found to have compound heterozygous mutations of *UNC13D* and reduced NK cell cytotoxic function (135). Soon after, variations in *UNC13D* were identified in 3/18 patients with s-JIA/MAS of which two had biallelic mutations (136). Starting from this, Vastert et al. sequenced *PRF1* in 54 s-JIA patients: 11 of 56 (20%) patients were heterozygous for missense mutations in *PRF1*, and s-JIA patients with history of MAS presented an increased prevalence of A91V mutation (20%) compared with s-JIA patients without history of MAS (9.8%) (137). Recurrent MAS was recently associated with monoallelic W374X mutation in *PRF1* in a child with s-JIA (111).

#### Other associations

Mutations in the *PRF1* gene were also detected in one 4-year-old girl with primary necrotizing lymphocytic CNS vasculitis (138) and in an 11-year-old girl with panniculitis. They were biallelic in the first case, monoallelic in the second one (139). Pasqualini et al. recently reported an 11-year-old boy with a history of secondary HLH who developed cytophagic histiocytic panniculitis, in whom mutation analysis showed monoallelic missense mutation of the *STX11* gene (140).

The investigation of *PRF1* in type I diabetes showed that allelic frequency of N252S was significantly higher in patients than in controls (141).

X-linked inhibitor of apoptosis protein mutations have been proposed to be involved in other phenotypes. Weiss et al. proposed *XIAP* as a putative modifier gene in Wilson disease (142). Ferretti et al. suggested a role of *XIAP* variants as a predisposing factor for idiopathic periodic fever (IPF) development, possibly through its influence on monocyte function. They evidenced a polymorphism, P423Q, at higher frequency in a large cohort of IPF patients. They also demonstrated that 423Q allele, as compared with 423P, was associated with higher Xiap protein and messenger RNA expression and lower caspase 9 activation (143). Recently, Ou et al. genotyped the *XIAP* Q423P polymorphism in 100 pediatric patients diagnosed with HLH and found its frequency to be comparable in patients and healthy controls (144).

Although association studies should always be taken with caution, unless investigation documents its pathogenic value in this specific condition, taken together these data indicate that sometimes different phenotypes can be associated to monoallelic or presumably “hypomorphic” mutations of FHL-related genes. In particular, a partial defect of granule-dependent cytotoxicity appears to be involved in the predisposition to cancer and autoimmunity, suggesting a link between these different pathogenic pathways and, likely, the role of other yet unknown genes.

## Contribution of FHL Patients to the Clarification of the Cytotoxic Machinery

**Figure 2:**
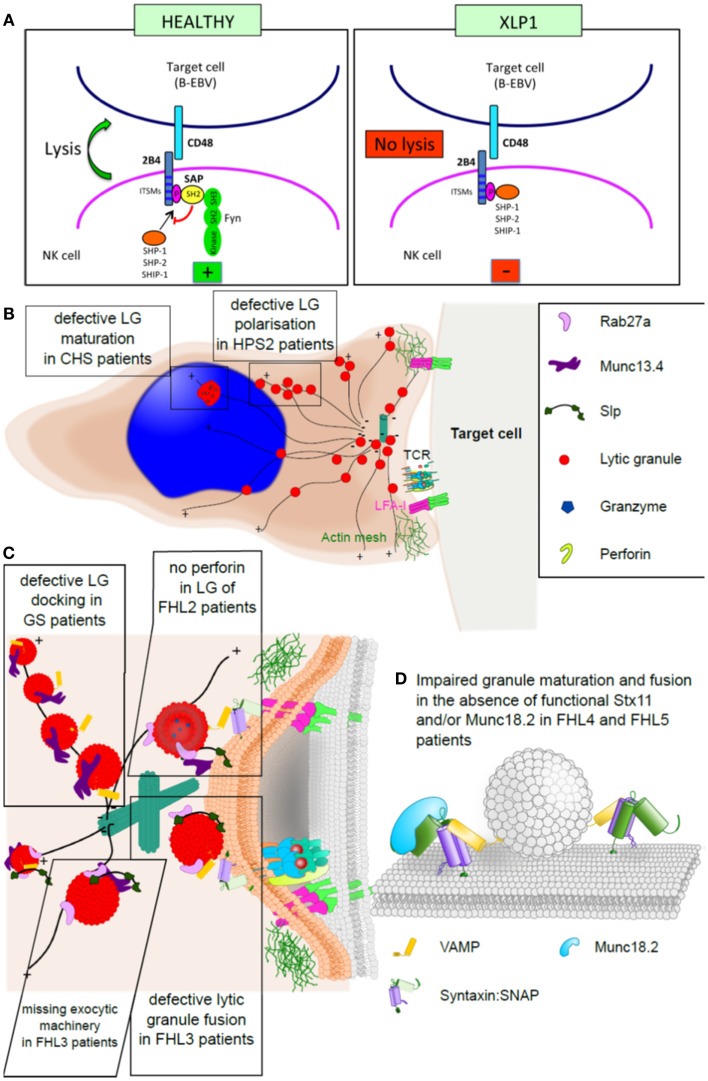
**Familial hemophagocytic lymphohistiocytosis and the immunological synapse**. **(A)** Simplified model of an NK cell synapse formed by a healthy (left) or SAP-deficient (right) cell. In a healthy NK cell, SAP binds to the cross-linked and phosphorylated 2B4 receptor, which leads to the recruitment of Fyn and consequently lysis of the target cell. In the absence of SAP, 2B4 associates with protein tyrosine phosphatases (SHP-1, SHP-2, and SHIP) delivering inhibitory signals. **(B)** Cartoon of a CTL synapse to illustrate the granule maturation and polarization defect observed in CHS and HPS2 patients, respectively. **(C)** Magnification of the CTL: target synapse area from **(B)**, depicting the docking defect observed in GS2 patients, the molecular function of Munc13.4 in granule maturation and priming, which is impaired in FHL3 patients, and lack of perforin in the granules of FHL2 patients. **(D)** Model illustrating the fusion of membranes by the activity of SNARE proteins (such as syntaxin 11) and Munc18-2, which is lost in FHL4 and FHL5 patients.

In the granule-dependent exocytosis pathway, target cell recognition by CTL and NK cells is followed by the polarized release of preformed cytolytic granules into the synaptic cleft formed between the effector and the target. Exocytosis of mature cytolytic granules from CTL and NK cells is a complex phenomenon and key for the clearance of virally infected or tumorigenic cells. The process that leads to fusion of cytolytic granules at the immunological synapse can be divided into four distinct steps: polarization, docking, priming, and fusion of cytolytic granules. The analysis of patient CTL and NK cells, the morphological comparison to cells from healthy individuals, assessing their ability to form cytotoxic granules and to polarize and secrete these has been critical to gain an insight into the molecular machinery that drives CTL and NK cell cytotoxicity. Only by careful analysis of patient CTL and NK cells we can now draw a molecular picture of how mutations in proteins hamper normal CTL and NK cell function (Figure 2).

Mutations in the small cytoplasmic signaling adaptor protein SAP (XLP-1) affect activation of NK cells. SAP associates with members of the SLAM family of trans-membrane receptors, including SLAM (CD150), LY9 (CD229), CRACC (CD319), CD84, NTB-A (CD352), and 2B4 (CD244) (145). 2B4 is a co-receptor expressed in NK and T lymphocytes and specifically recognizes CD48, which is present solely on hematopoietic cells. 2B4 engagement causes tyrosine phosphorylation of immune receptor tyrosine-based switch motifs (ITSM), recruitment of SAP and thus transduction of activating signals via Fyn-dependent processes (145, 146). In the absence of functional SAP, 2B4 associates with protein tyrosine phosphatases and delivers inhibitory signals (53, 55, 147). Consequently, lack of NK cell-mediated cytotoxicity in XLP-1 mainly stems from the inhibitory effect of 2B4, which, upon engagement by CD48 (up-regulated on B-EBV cells), impairs the function of activating receptors (52). Beyond their common chromosomal localization and their requirement for normal immune responses to certain viral infections, SAP and XIAP showed no structural or functional similarity, are not co-regulated, and do not appear to directly interact (65). SAP functions as an intracellular adaptor molecule involved in SLAM family signaling (51). XIAP is an inhibitor of apoptosis family member, known for its caspase-inhibitory and anti-apoptotic properties. In patients with SAP deficiency, HLH may develop for several reasons including absence of iNKT cells and defective T-cell reactivation-induced cell death (148–150). The mechanism involved in XIAP deficient patients is still unclear.

Activation and priming of NK and CTL leads to the formation of secretory lysosomes, which contain lytic molecules including perforin, granzymes (a family of serine proteases), granulysin, and other lysosomal enzymes, but also a proteoglycan matrix (serglycin) that maintains proteases in an inactive stage, perforin inhibitor (calreticulin), and Fas ligand. CTL from CHS patients, however, show enlarged lysosomal compartments with ER-specific membrane proteins and autophagic inclusions, indicating a function of Lyst during lysosomal fission (151, 152). Why these enlarged lytic granules fail to fuse is still not fully understood. It could be that the structures are simply too big to polarize and fuse.

Similar to the function of Lyst, the AP-3 complex is also required for proper lytic granule maturation in CTL. Defects in the β-3A subunit disrupt the entire AP-3 complex, lead to lysosomal protein mis-sorting in some cell types including melanosomes and platelets, as well as CTLs and NK cells (153, 154). In melanocytes AP-3 sorts tyrosinase and melanin-processing enzymes into melanosomes (155). However, the exact molecular function of AP-3 during secretory lysosome maturation in CTL and NK cells is not yet fully understood. Phenotypically, HPS2 patient cells show an increase in tubular–vesicular endosomes and secretory lysosomes that fail to polarize to the IS (156, 157). These observations suggest a role of AP-3 in the trafficking of a microtubule motor protein or its adaptor to secretory lysosomes (80). Interestingly, most HPS2 patients only show a mild HLH phenotype posing the question whether residual amounts of functional AP-3 are sufficient to sustain CTL and NK cell function, or whether loss of AP-3 may be compensated for by a different adaptor protein (157).

Once polarized, secretory lysosomes in healthy CTL dissociate from the microtubules and dock at the immunological synapse. In Rab27a-deficient GS2 patient CTL secretory lysosomes polarize normally at the immunological synapse but then remain attached to the microtubules, resembling beads on a string (158), providing evidence that Rab27a facilitates granule detachment and docking. Interestingly, loss of Munc13-4 from CTL impairs secretion at a similar stage; however, in contrast to GS2, secretory lysosomes in Munc13-4-deficient CTL detach from the microtubules and dock, but fail to fuse with the plasma membrane (95, 158). Taken together, these morphological phenotypes suggest that Rab27a and Munc13-4 function in consecutive steps during cytotoxicity, by mediating lytic granule docking and priming, respectively; a hypothesis strengthened by the finding that Rab27a and Munc13-4 interact directly (159–162).

As a member of the Rab family, the small guanine nucleotide-binding protein Rab27a operates as a molecular switch, and by that can controls a myriad of cellular processes. Besides the interaction with Munc13-4, active Rab27a also binds a class of proteins that is closely related to neuronal synaptotagmin I: the synaptotagmin-like proteins (Slps) (163). In melanocytes, Rab27a binds the synaptotagmin-like protein melanophilin and the plus-end directed actin motor myosin Va (164–166). This complex facilitates the polarization and docking of melanosomes at the cell’s periphery and explains why loss of functional Rab27a causes albinism. In CTL, however, active Rab27a appears to interact with various, functionally redundant Slps (namely Slp1, Slp2a, and Slp3) since only over-expression of a dominant-negative construct against the highly conserved SHD domain of these Slps resulted in a striking cytotoxicity phenotype (165, 167–169). It is conceivable that Slp1, Slp2a, and Slp3 act as vesicle tethers at the plasma membrane or form the linker between Rab27a and a motor protein.

Munc13-4 is one of four mammalian homologs of *C. elegans* Unc13 (170). Munc13 proteins vary significantly in size and in the number of functional domains; however, the C-termini of all Munc13 proteins are highly homologous, encoding the recently defined MUN domain (171). One role fulfilled by Munc13-4 is that of a vesicle tether, arresting the movement of Rab27a-positive vesicles at the site of secretion and enabling vesicle priming (161, 172). This function is most likely exerted by the central MUN domain, due to its similarity to vesicle tethering proteins Sec6p and Vps53 (173–175). Moreover, Munc13-4 was recently shown to interact with the SNARE domain of Stx11 and t-SNARE complexes, thus offering a mechanism by which Munc13-4 could mediate vesicle priming and initiate fusion (176).

Besides its role as tether/priming component, which depends on the interaction with Rab27a, Munc13-4 has also been connected to lytic granule maturation in a Rab27a-independent mechanism by facilitating fusion between two distinct endosomal compartments (162). This model suggests that cytolytic granules in Munc13-4-deficient CTL lack the necessary fusion machinery, explaining why vesicle release is arrested after docking.

The final step during cytotoxicity is the release of cytotoxic mediators into the cleft between CTL and the target cell; a process that is facilitated by SNARE proteins, as well as SNARE accessory proteins (177). Stx11 is an unusual member of the syntaxin family of SNARE proteins as it associates with membranes through a cysteine-rich region at its C terminus, instead of the C-terminal trans-membrane domain (178). It still remains elusive whether lipid anchored SNARE proteins can facilitate membrane fusion, with reports arguing both ways (178–181). It is conceivable that Stx11 may form inhibitory, fusion incapable SNARE complex, potentially with Vti1b, and thus regulate membrane fusion indirectly. However, the observation that Stx11 can also form a SNARE complex with SNAP23 and VAMP8, which itself is crucial for CTL cytotoxicity (34, 182), supports an active role of Stx11 during membrane fusion.

Much of the molecular function of Stx11 depends on Munc18-2, another critical component of the CTL fusion machinery. One of the functions of Munc18-2 is to chaperone Stx11 as shown by reduced protein levels of Stx11 in Munc18-2-deficient CTL (40, 41). This role depends on the direct interaction between Munc18-2 and Stx11 and is most likely initiated and selected for by the N peptide of Stx11. Strikingly, the killing defect observed in CTL and NK cells from FHL4 and FHL5 patient can often be compensated for by *in vitro* activation with IL-2 (40, 41, 43, 46). This may be partially due to the up-regulation of surrogate proteins, namely Stx3 and Munc18-1. Interestingly, in the absence of Stx11 Stx3 is trafficked to the plasma membrane in a Munc18-2-dependent manner, adding evidence to the model that Stx11 is indeed involved in the final fusion event (183). Besides the chaperone function, detailed analysis of reported mutations additionally concluded that Munc18-2 will most likely function during SNARE complex formation, binding and fusion itself, similar to the role of its homolog Munc18-1 in neurons (184–186).

## Conclusion

Familial hemophagocytic lymphohistiocytosis is a rare, life-threatening disease with a non-specific clinical presentation, which needs accurate clinical, immunological, and genetic diagnostic work-up. Current standard of therapy based on chemo-immunotherapy (dexamethasone and etoposide) allows rapid disease control in most but not all patients. Due to the remaining risk of early mortality, novel therapeutic approaches based on the use of anti-thymocyte globulin (ATG or anti-CD52/Campath), or an anti-IFN-γ human monoclonal antibody, are currently explored in international cooperative settings. Currently identified genetic defects allow the assignment of a genetic marker to over 80% of the families, with proportions varying according to the geographic areas. In the remaining patients, some cases show either familial recurrence and/or the disease is refractory or recurs after initial treatment; these items strongly suggest an additional genetic defect beyond the ones we currently investigate. All such cases should be addressed to reference laboratories with research capabilities, where the combined use of confocal microscopy, cellular cytotoxicity assays, and protein expression studies may help to better characterize putative novel defects. In turn, those studies will broaden our current knowledge of the cellular cytotoxic machinery in humans. In the meanwhile, observations of mono or even biallelic mutations in FHL-related genes in patients with phenotypes different from FHL, may provide additional insights into our understanding of the role of this part of innate and adaptive immunity.

## Conflict of Interest Statement

The authors declare that the research was conducted in the absence of any commercial or financial relationships that could be construed as a potential conflict of interest.
